# Author Correction: Anion-conducting channelrhodopsins with tuned spectra and modified kinetics engineered for optogenetic manipulation of behavior

**DOI:** 10.1038/s41598-018-23001-5

**Published:** 2018-03-14

**Authors:** Jonas Wietek, Silvia Rodriguez-Rozada, Janine Tutas, Federico Tenedini, Christiane Grimm, Thomas G. Oertner, Peter Soba, Peter Hegemann, J. Simon Wiegert

**Affiliations:** 1 Institute for Biology, Experimental Biophysics, Humboldt-Universität zu Berlin, Invalidenstraße 42, 10115 Berlin, Germany; 2 Research Group Neuronal Patterning and Connectivity, Center for Molecular Neurobiology Hamburg, Falkenried 94, 20251 Hamburg, Germany; 3 Institute for Synaptic Physiology, Center for Molecular Neurobiology Hamburg, Falkenried 94, 20251 Hamburg, Germany; 4 Research Group Synaptic Wiring and Information Processing, Center for Molecular Neurobiology Hamburg, Falkenried 94, 20251 Hamburg, Germany

Correction to: *Scientific Reports* 10.1038/s41598-017-14330-y, published online 02 November 2017

This Article contains errors in the Results section under subheading ‘Biophysical characterization in HEK cells’.

“Because Chronos exhibits no serine at the homologous position of S63, which is a main constituent of the inner gate in *Cr*ChR2 we speculated that a differently arranged inner gate could be responsible for the missing photocurrents. Therefore, we created the double mutant Chronos^A80S E107R^ to reconstitute a serine residue at the putative inner gate while rendering the central gate anion conductive.”

should read:

“Because Chronos exhibits no serine at the homologous position of S63, which is a main constituent of the central gate in *Cr*ChR2 we speculated that a differently arranged central gate could be responsible for the missing photocurrents. Therefore, we created the double mutant Chronos^A80S E107R^ to reconstitute a serine residue at the putative central gate while rendering the central gate anion conductive.”

