# Supplementary material for: Anion-conducting channelrhodopsins with tuned spectra and modified kinetics engineered for optogenetic manipulation of behavior
Source: Sci Rep. 2017 Nov 2;7:14957. doi: 10.1038/s41598-017-14330-y (PMC5668261; doi:10.1038/s41598-017-14330-y)
Supplement: Supplementary file 8 — Supplementary information [file 41598_2017_14330_MOESM8_ESM.pdf]

Supplementary Material for:

Title:

Anion-conducting channelrhodopsins with tuned spectra and modified kinetics engineered for optogenetic manipulation of behavior

Authors:

Jonas Wietek[1], Silvia Rodriguez-Rozada[4], Janine Tutas[2], Federico Tenedini[2], Christiane Grimm[1], Thomas G. Oertner[3], Peter Soba[2], Peter Hegemann[1], J. Simon Wiegert[3,4] *

Affiliations:

1 Institute for Biology, Experimental Biophysics, Humboldt-Universität zu Berlin, Invalidenstraße 42, 10115 Berlin, Germany.

2 Research Group Neuronal Patterning and Connectivity, Center for Molecular Neurobiology Hamburg, Falkenried 94, 20251 Hamburg, Germany.

3 Institute for Synaptic Physiology, Center for Molecular Neurobiology Hamburg, Falkenried 94, 20251 Hamburg, Germany.

4 Research Group Synaptic Wiring and Information Processing, Center for Molecular Neurobiology Hamburg, Falkenried 94, 20251 Hamburg, Germany

* correspondence to: simon.wiegert@zmnh.uni-hamburg.de

```
                                                                                                                       H1
iChloC          ------------------------------MDYG------------------G---ALSAVGRELLFVTN-PVVVNGSV--LVP-------EDQCYCAGWIESRGTNGAQTASNVLQWLA  59
iC++            -----------------------------------------------------MDYG-GALSAVGLFQTSYTLENNGSVICIPN-------NGQCFCLAWLKSNGTNAEKLAANILQWIS  59
Phobos          -----------------------------------------------------MDYG-GALSAVGLFQTSYTLENNGSVICIPN-------NGQCFCLAWLKSNGTNAEKLAANILQWIS  59
Aurora          -----------------------------------------------------MDYG-GALSAVGLFQTSYTLENNGSVICIPN-------NGQCFCLAWLKSNGTNAEKLAANILQWVS  59
TcChR           --------------------------------------------------------------MGWKINPLYSDEVAILEI----------------CKENEMVFGPLWEQKLARALQWFT  42
TsChR           ---------------------------------------------------------------MFAINPEYMNETVLLDE----------------CTPIYLDIGPLWEQVVARVTQWFG  41
PsChR2          --------------------------------------------------------------MGFQLNPEYLNETILLDD----------------CTPIYLNVGPLWEQKVARGTQWFG  42
CrChR2          ------------------------------MDYG------------------G---ALSAVGRELLFVTN-PVVVNGSV--LVP-------EDQCYCAGWIESRGTNGAQTASNVLQWLA  59
CoChR           ------------------------------------------------------------------------MLGNGSA--IVP-------IDQCFCLAWTDSLGSDTEQLVANILQWFA  39
C1C2            ----MS-----------RRPWLLALALAVALAAGSAGASTGSDATVPVATQDGPDYVFHRAHERMLFQTSYTLENNGSVICIPN-------NGQCFCLAWLKSNGTNAEKLAANILQWIT  98
ShChR(Chronos)  ---MET-------AATMTHAFISAVPSAEAT---IRGLLSAAAVVTPAADAHG-----------E----TSNATTAGADHGCFPH-----------------INHGTELQHKIAVGLQWFT  76
VcChR1          -----------------------------------------------------MDYPVAR-----SLIVRYPTDLGNGTVCMP--------RGQCYCEGWLRSRGTSIEKTIAITLQWVV  54
C1V1b           ----MS-----------RRPWLLALALAVALAAGSAGASTGSDATVPVATQDGPDYVFHRAHERMLFQTSYTLENNGSVICIPN-------NGQCFCLAWLKSNGTNAEKLAANILQWIT  98
ReaChR          ---MVS-----------RRPWLLALALAVALAAGSAGASTGSDATVPVATQDGPDYVFHRAHERMLFQTSYTLENNGSVICIPN-------NGQCFCLAWLKSNGTNAEKLAANILQWVV  99
bReaChES        -----------------------------------------------------MDYG-GALSAVGLFQTSYTLENNGSVICIPN-------NGQCFCLAWLKSNGTNAEKLAANILQWVV  59
CnChR1          MAELISSATRSLFAAGGINPWPNPYHHE---DMGCG-------GMTPTGECF---------STEWWCDPSYGLSDAGYGYCFVEATGGYLVVGVEKKQAWLHSRGTPGEKIGAQVCQWIA  101

                   H1                       H2                                  H3                                H4         H5
iChloC          AGFSILLLMFYAYQTWKSTCGWEQIYVCAIRMVKVILEFFFSFKNPSMLYLATGHRVQWLRYAEWLLTCPVILIHLSNLTGLSNDYSRRTMGLLVSNIGCIVWGATSAMATGYVKVIFFC  179
iC++            FALSALCLMFYGYQTWKSTCGWENIYVATIQMIKFIIEYFHSFDEPAVIYSSNGNKTRWLRYASWLLTCPVILIHLSNLTGLANDYNKRTMGLLVSDIGTIVWGTTAALSKGYVRVIFFL  179
Phobos          FALSALCLMFYGYQTWKSTCGWENIYVATIQMIKFIIEYFHSFDEPAVIYSSNGNKTRWLRYASWLLTCPVILIHLSNLTGLANDYNKRTMGLLVSDIGGIVWATTAALSKGYVRVIFFL  179
Aurora          FALSVACLGWYAYQAWRATCGWENVYVALIQMMKSIIEAFHSFDSPATLWLSSGNGVRWMRYGSWLLTCPVILIHLSNLTGLKDDYSKRTMGLLVSDVGCIVWGATSAMCTGWTKILFFL  179
TcChR           VILSAIFLAYYVYSTLRATCGWEELYVCTVEFTKVVVEVYLEYVPPFMIYQMNGQHTPWLRYMEWLLTCPVILIHLSNITGLNDEYSGRTMSLLTSDLGGIAFAVLSALAVGWQKGLYFG  162
TsChR           VILSLVFLIYYIWNTYKATCGWEELYVCTVEFCKIIIELYFEYTPPAMIFQTNGQVTPWLRYSEWLLTCPVILIHLSNITGLNDDYSGRTMSLITSDLGGICMAVTAALSKGWLKALFFV  161
PsChR2          VILSLAFLIYYIWITYKATCGWEELYVCTIEFCKIVIELYFEFSPPAMIYQTNGEVTPWLRYAEWLLTCPVILIHLSNITGLNDDYSGRTMSLITSDLGGICMAVTSALSKGWLKWLFFV  162
CrChR2          AGFSILLLMFYAYQTWKSTCGWEEIYVCAIEMVKVILEFFFEFKNPSMLYLATGHRVQWLRYAEWLLTCPVILIHLSNLTGLSNDYSRRTMGLLVSDIGTIVWGATSAMATGYVKVIFFC  179
CoChR           FGFSILILMFYAYQTWRATCGWEEVYVCCVELTKVIIEFFHEFDDPSMLYLANGHRVQWLRYAEWLLTCPVILIHLSNLTGLKDDYSKRTMRLLVSDVGTIVWGATSAMSTGYVKVIFFV  159
C1C2            FALSALCLMFYGYQTWKSTCGWEEIYVATIEMIKFIIEYFHEFDEPAVIYSSNGNKTVWLRYAEWLLTCPVILIHLSNLTGLANDYNKRTMGLLVSDIGTIVWGTTAALSKGYVRVIFFL  218
ShChR           VIVAIVQLIFYGWHSFKATTGWEEVYVCVIELVKCFIELFHEVDSPATVYQTNGGAVIWLRYSMWLLTCPVILIHLSNLTGLHEEYSKRTMTILVTDIGNIVWGITAAFTKGPLKILFFM  196
VcChR1          FALSVACLGWYAYQAWRATCGWEEVYVALIEMMKSIIEAFHEFDSPATLWLSSGNGVVWMRYGEWLLTCPVLLIHLSNLTGLKDDYSKRTMGLLVSDVGCIVWGATSAMCTGWTKILFFL  174
C1V1b           FALSALCLMFYGYQTWKSTCGWEEIYVATIEMIKFIIEYFHEFDEPAVIYSSNGNKTVWLRYAEWLLTCPVLLIHLSNLTGLKDDYSKRTMGLLVSDVGCIVWGATSAMCTGWTKILFFL  218
ReaChR          FALSVACLGWYAYQAWRATCGWEEVYVALIEMMKSIIEAFHEFDSPATLWLSSGNGVVWMRYGEWLLTCPVILIHLSNLTGLKDDYSKRTMGLLVSDVGCIVWGATSAMCTGWTKILFFL  219
bReaChES        FALSVACLGWYAYQAWRATCGWEEVYVALIEMMKSIIEAFHEFDSPATLWLSSGNGVVWMRYGSWLLTCPVILIHLSNLTGLKDDYSKRTMGLLVSDVGCIVWGATSAMCTGWTKILFFL  179
CnChR1          FSIAIALLTFYGFSAWKATCGWEEVYVCCVEVLFVTLEIFKEFSSPATVYLSTGNHAYCLRYFEWLLSCPVILIKLSNLSGLKNDYSKRTMGLIVSCVGMIVFGMAAGLATDWLKWLLYI  221

                         H5                          H6                                   H7
iChloC          LGLCYGANTFFHAAKAYIEGYHTVPKGRCRQVVTGMAWLFFVSWGMFPILFILGPEGFGVLSVYGSTVGHTIIDLMSKNCWGLLGHYLRVLIHEHILIHGDIRKTTKLNIGGTEIEVETLVEDEAEAGAV------  309
iC++            MGLCYGIYTFFNAAKVYIEAYHTVPKGRCRQVVTGMAWLFFVSWGMFPILFILGPEGFGVLSRYGSNVGHTIIDLMSKQCWGLLGHYLRVLIHSHILIHGDIRKTTKLNIGGTEIEVETLVEDEAEAGAV------  309
Phobos          MGLCYGIYTFFNAAKVYIEAYHTVPKGRCRQVVTGMAWLFFVSWGMFPILFILGPEGFGVLSRYGSNVGHTIIDLMSKQCWGLLGHYLRVLIHSHILIHGDIRKTTKLNIGGTEIEVETLVEDEAEAGAV------  309
Aurora          ISLSYGMYTYFHAAKVYIEAFHTVPKGLCRQLVRAMAWLFFVSWGMFPVLFLLGPEGFGHISRYGSNIGHSILDLIAKQMWGVLGNYLRVKIHSHILLYGDIRKKQKITIAGQEMEVETLVAEEED----------  305
TcChR           IGCIYGASTFYHAACIYIESYHTMPAGKCKRLVVAMCAVFFTSWFMFPALFLAGPECFDGLTWSGSTIAHTVADLLSKNIWGLIGHFLRVGIHEHILVHGDVRRPIEVTIFGKETSLNCFVENDDEEDDV------  292
TsChR           IGCGYGASTFYNAACIYIESYYTMPQGICRRLVLWMAGVFFTSWFMFPGLFLAGPEGTQALSWAGTTIGHTVADLLSKNAWGMIGHFLRVEIHKHIIIHGDVRRPVTVKALGRQVSVNCFVDKEEEEEDERI----  293
PsChR2          IGCCYGASTFYHAALIYIESYYTMPHGVCKNMVLAMAAVFFTSWFMFPGLFLAGPEGTNALSWAGSTIGHTVADLLSKNAWGMIGHFLRLEIHKHIIIHGDVRRPITVNTLGREVTVSCFVDKEEEDEDERA----  294
CrChR2          LGLCYGANTFFHAAKAYIEGYHTVPKGRCRQVVTGMAWLFFVSWGMFPILFILGPEGFGVLSVYGSTVGHTIIDLMSKNCWGLLGHYLRVLIHEHILIHGDIRKTTKLNIGGTEIEVETLVEDEAEAGAV------  311
CoChR           LGCIYGANTFFHAAKVYIESYHVVPKGRPRTVVRIMAWLFFLSWGMFPVLFVVGPEGFDAISVYGSTIGHTIIDLMSKNCWGLLGHYLRVLIHQHIIIYGDIRKKTKINVAGEEMEVETMVDQEDEETV-------  288
C1C2            MGLCYGIYTFFNAAKVYIEAYHTVPKGRCRQVVTGMAWLFFVSWGMFPILFILGPEGFGVLSVYGSTVGHTIIDLMSKNCWGLLGHYLRVLIHEHILIHGDIRKTTKLNIGGTEIEVETLVEDEAEAGAV------  348
ShChR           IGLFYGVTCFFQIAKVYIESYHTLPKGVCRKICKIMAYVFFCSWLMFPVMFIAGHEGLGLITPYTSGIGHLILDLISKNTWGFLGHHLRVKIHEHILIHGDIRKTTTINVAGENMEIETFVDEEEEGGV-------  325
VcChR1          ISLSYGMYTYFHAAKVYIEAFHTVPKGICRELVRVMAWTFFVAWGMFPVLFLLGTEGFGHISPYGSAIGHSILDLIAKNMWGVLGNYLRVKIHEHILLYGDIRKKQKITIAGQEMEVETLVAEEEDDTVKQSTAKY  310
C1V1b           ISLSYGMYTYFHAAKVYIEAFHTVPKGICRELVRVMAWTFFVAWGMFPVLFLLGTEGFGHISPYGSAIGHSILDLIAKNMWGVLGNYLRVKIHEHILLYGDIRKKQKITIAGQEMEVETLVAEEED----------  344
ReaChR          ISLSYGMYTYFHAAKVYIEAFHTVPKGLCRQLVRAMAWLFFVSWGMFPVLFLLGPEGFGHISPYGSAIGHSILDLIAKNMWGVLGNYLRVKIHEHILLYGDIRKKQKITIAGQEMEVETLVAEEEDKYESS-----  350
bReaChES        ISLSYGMYTYFHAAKVYIEAFHTVPKGLCRQLVRAMAWLFFVSWGMFPVLFLLGPEGFGHISPYGSAIGHSILDLIAKNMWGVLGNYLRVKIHEHILLYGDIRKKQKITIAGQEMEVETLVAEEED----------  305
CnChR1          VSCIYGGYMYFQAAKCYVEANHSVPKGHCRMVVKLMAYAYFASWGSYPILWAVGPEGLLKLSPYANSIGHSICDIIAKEFWTFLAHHLRIKIHEHILIHGDIRKTTKMEIGGEEVEVEEFVEEEDEDTVAA-----  352
```

Figure S1: Sequence comparison of eACRs and CCRs. Amino acid sequence alignment[1-3] of iChloC (dark gray), iC++ (dark turquoise), Phobos (deep purple), Aurora (dark red) and all cation-conducting ChRs (CCRs) mutated in this study. Mutations yielding the four ACRs are indicated with bold red letters and homologous positions in CCRs are shown in green. In addition, the E90R (bold orange) as well as E83Q and E101S (bold pink) mutations are highlighted in the iChloC sequence. C128, which was mutated to A to generate SFO-ACRs is highlighted in gray. T159G and G163A mutations in blue-shifted opsins are highlighted in purple. The N-termini replaced for the N-terminus of iC++ (dark bold turquoise) are shown in gray. Please note that the Aurora sequence is identical to bReachES[4] except for the introduced mutations. Crossed amino acids where left out for iC++ based approach.

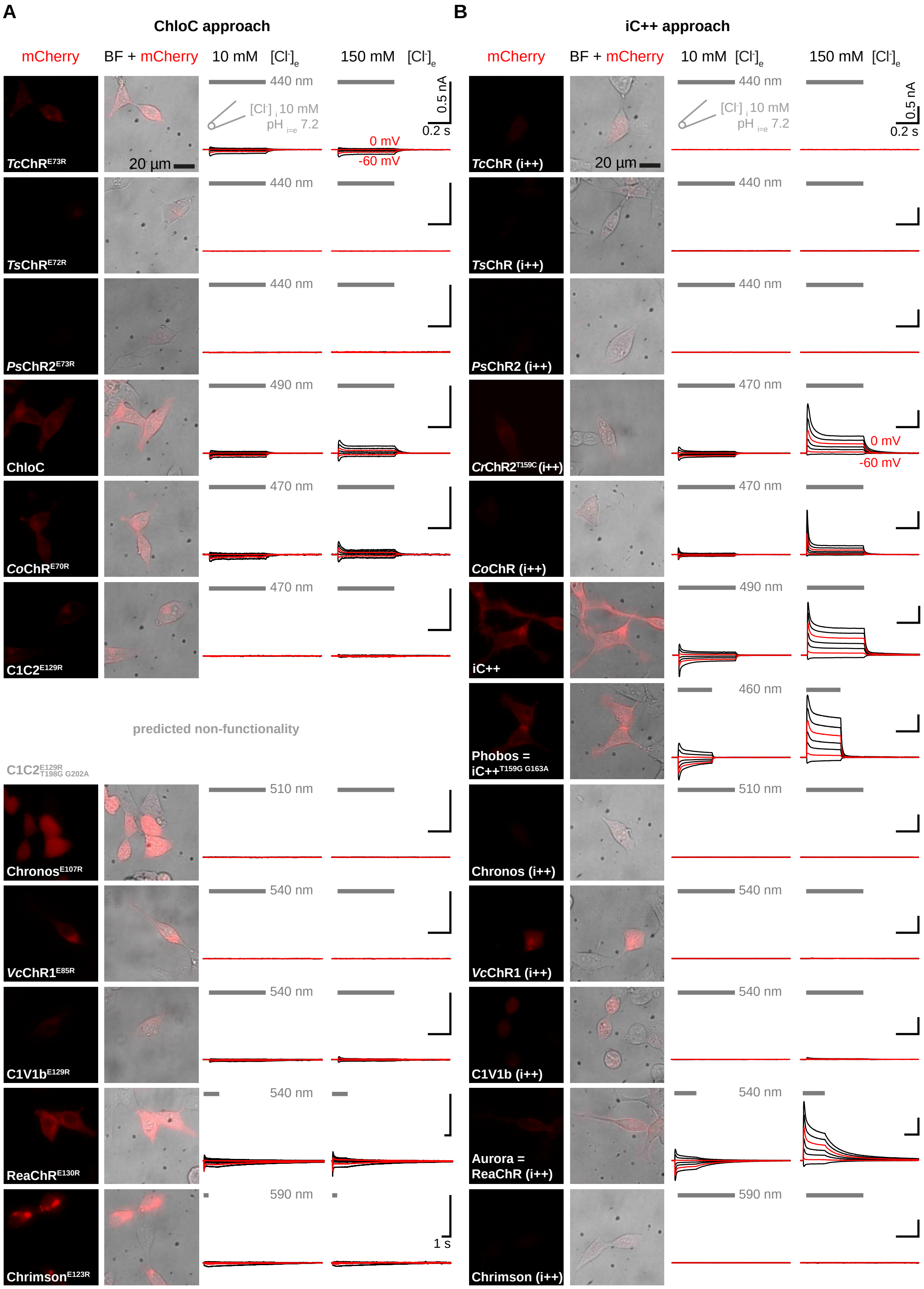

Figure S2: Screening results of the conversion approaches. Summary of converted CCRs tested in this study. Widefield epifluorescence images show localization of converted CCRs labeled by mCherry in HEK cells. Corresponding brightfield (BF) images show HEK cell morphology. Photocurrents at holding potentials ranging from -80 to +40 mV (20 mV increments) in low (10 mM, left) or high (150 mM, right) extracellular chloride. The ChloC-strategy is shown in (**A**), the iC++ approach is shown in (**B**). Parental CCRs with their mutations are indicated for all constructs. For CCR abbreviations please refer to the main text.

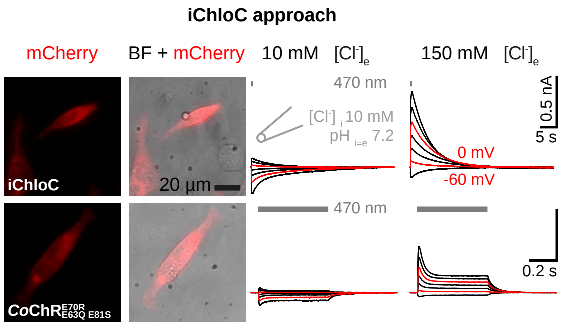

Figure S3: Characterization of *Cr*ChR2 and *Co*ChR converted with the iChloC approach. Widefield epifluorescence images show localization of converted CCRs labeled by mCherry in HEK cells. Corresponding brightfield (BF) images show HEK cell morphology. Photocurrents at holding potentials ranging from -80 to +40 mV (20 mV increments) in low (10 mM, left) or high (150 mM, right) extracellular chloride.

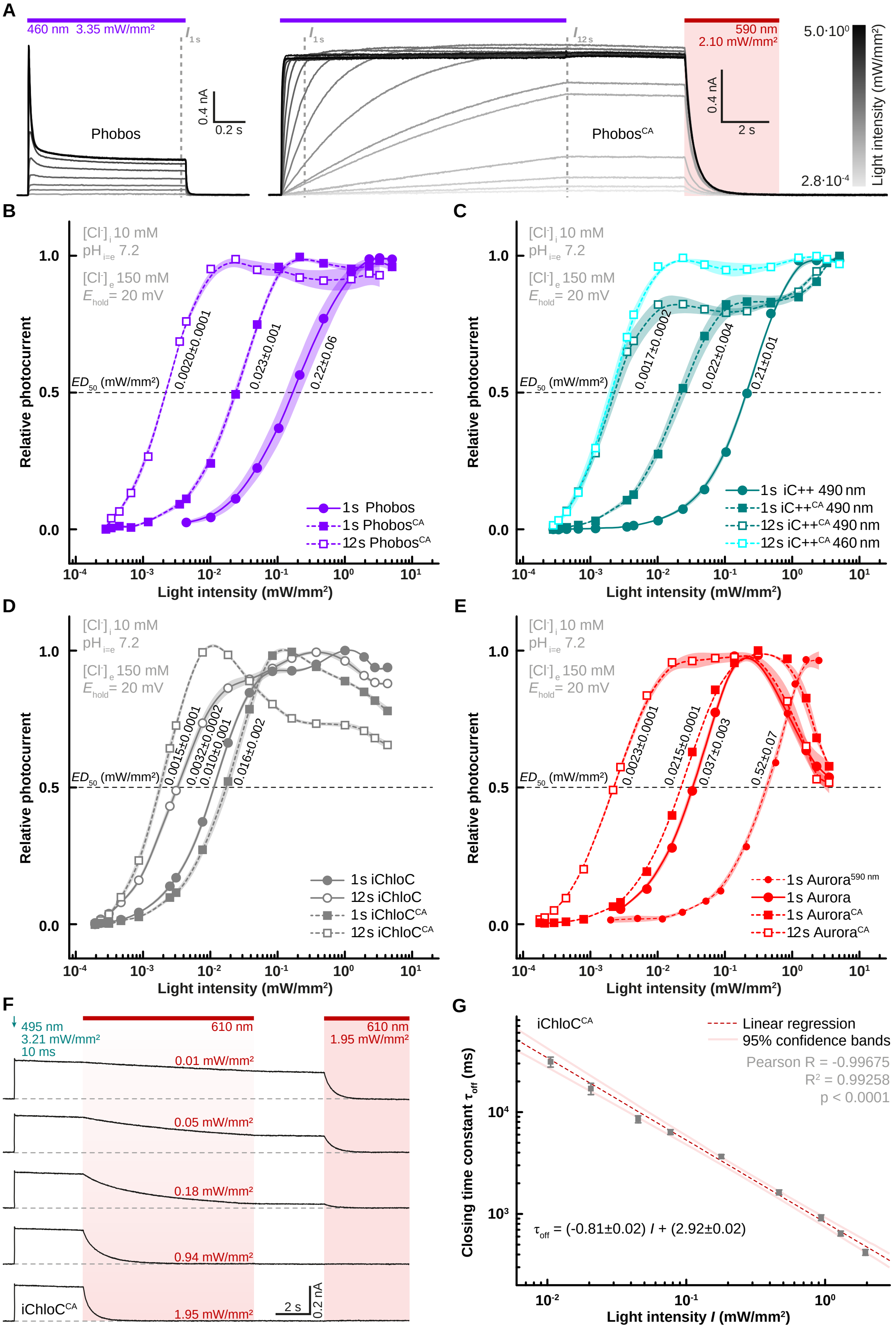

Figure S4: Biophysical properties of step-function-eACRs. (**A**) Typical photocurrent traces for Phobos (left) and its step-function variant Phobos^{CA} (right) obtained from light titration experiments. Light was applied for 1 s (Phobos) or 12 s (Phobos^{CA}). The holding potential was +20 mV. Analyzed currents are indicated by $I_{(time)}$ and gray dotted lines. Light intensities vary from bottom to top as indicated. For Phobos^{CA} channel closure was accelerated between sweeps with orange light. (**B** to **E**) Light titration curves obtained from measurements as shown in (A) for fast- and slow-cycling variants of Phobos (B), iC++(C), iChloC (D) and Aurora (E). The light intensities needed to obtain half maximal stationary photocurrents (ED_{50}) for different illumination durations (1 and 12 s) are plotted as mean ± SEM (n= 5 Phobos, 4 Phobos^{CA}, 7 iC++, 6 iC++^{CA}, 5 Aurora, 5 Aurora 590 nm, 4 Aurora^{CA}, 5 iChloC, 4 iChloC^{CA}). Light titration of iC++^{CA} (C) termed SwichR++ in[5] shows reduced partial inactivation at 460 nm (n= 3) compared to activation with 490 nm. (**F** and **G**) Dependence of accelerated iChloC^{CA} channel closing on light dose. (F) Example traces from a single experiment. (G) Data from multiple experiments (n= 6 HEK cells) show a linear relationship between closing speed and light intensity. Statistical parameters of fits are provided in the figure panel.

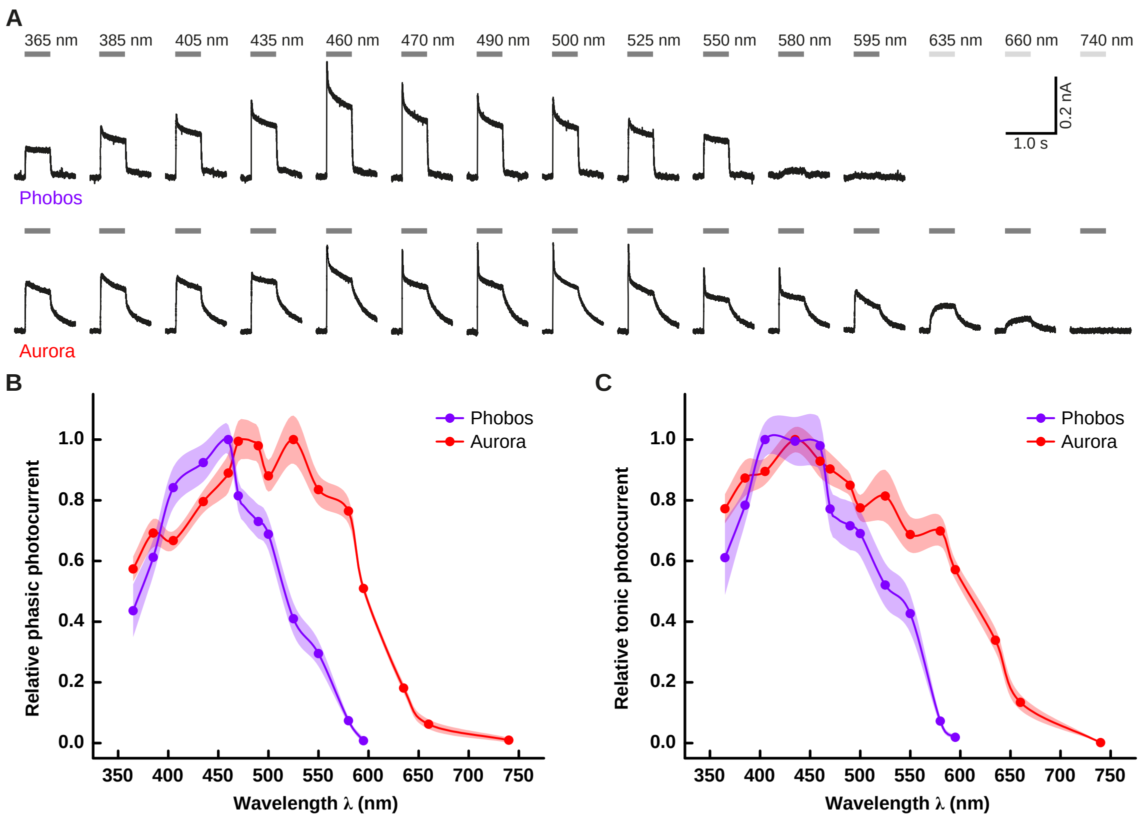

Figure S5: Action spectra of Phobos and Aurora photocurrents in neurons. (**A**) Example traces of Phobos (top row) or Aurora photocurrents (bottom row) evoked at indicated wavelengths (500 ms, 10 mW/mm^2) in CA1 pyramidal neurons clamped at -50 mV. (**B**) and (**C**) Action spectra of normalized peak (B) and tonic (C) Phobos and Aurora photocurrents.

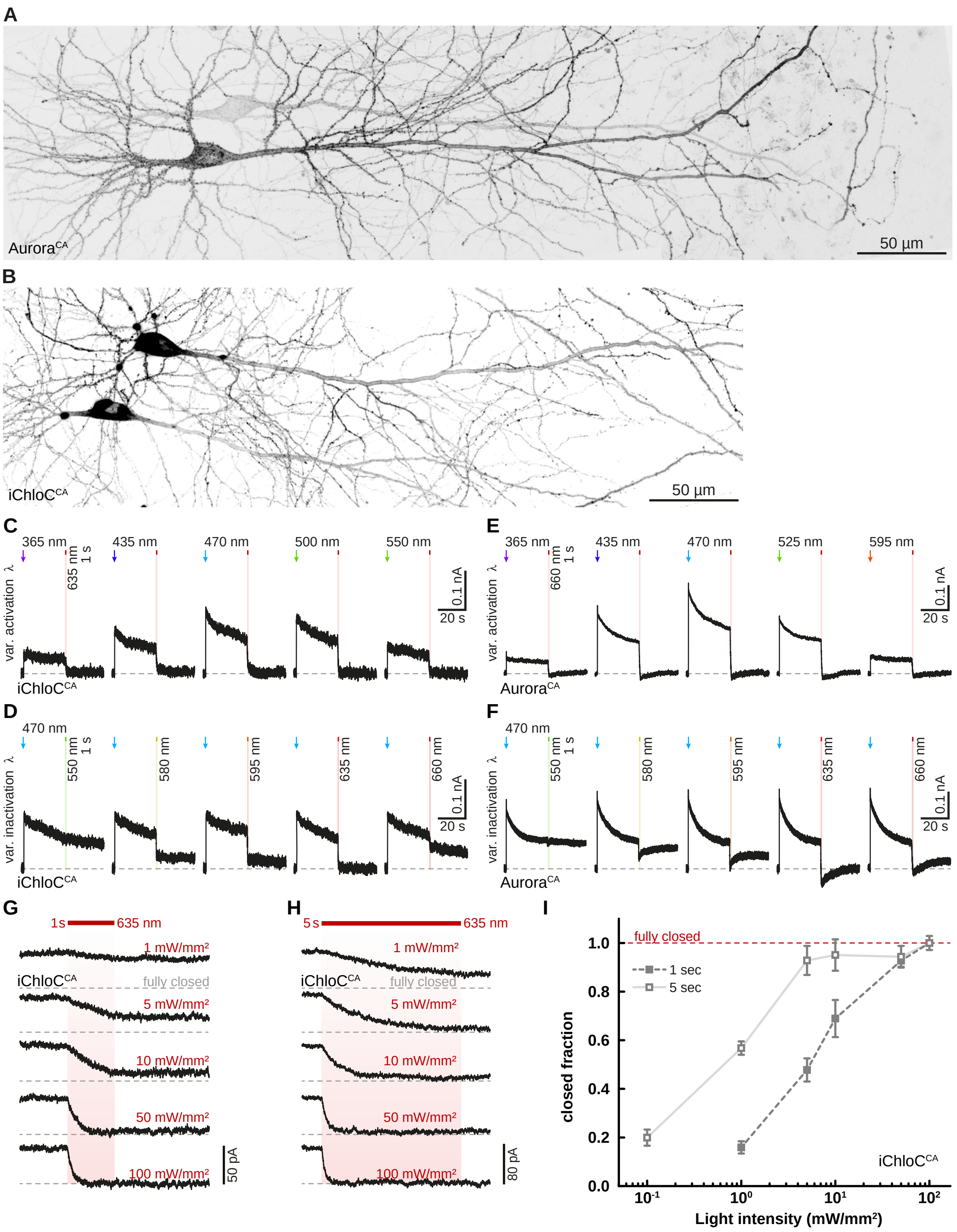

Figure S6: Light-dependent activation and inactivation of SFO-eACRs in CA1 pyramidal neurons. (**A** and **B**) CA1 pyramidal neurons expressing Aurora^{CA}-Citrine (A) or iChloC^{CA}-Citrine (B) 5 days after electroporation (stitched maximum intensity projections of two-photon images, fluorescence intensity shown as inverted gray values). Citrine fluorescence was mainly localized to the plasma membrane in Aurora^{CA} expressing cells. However, iChloC^{CA}-Citrine expressing neurons (B) showed additional, strong protein accumulation in the soma. (**C**) Representative recordings of photocurrents in an iChloC^{CA}-Citrine expressing CA1 cell. Photocurrents were evoked with different activation wavelengths and shutoff with 635 nm light. (**D**) Photocurrent traces in the same cell evoked with 470 nm light and shutoff at indicated wavelengths (10 mW/mm^2). (**E**) Representative recordings of photocurrents in an Aurora^{CA}-Citrine expressing CA1 cell. Photocurrents were evoked with different activation wavelengths and shutoff with 660 nm light. (**F**) Photocurrent traces in the same cell evoked with 470 nm light and shut off at indicated wavelengths (10 mW/mm^2). (**G** and **H**) Example recordings showing light-accelerated channel closing of iChloC^{CA}-Citrine in CA1 pyramidal neurons. Channel closing was accelerated by illumination with 635 nm light at indicated powers for 1 s (G) or 5 s (H). (**I**) Quantification of experiments shown in (G) and (H). Full channel closing could be achieved with 5 mW/mm^2 over 5 s or 50 mW/mm^2 over 1 s (n= 5 neurons in 5 slice cultures). Averages are shown as rectangular symbols ± SEM.

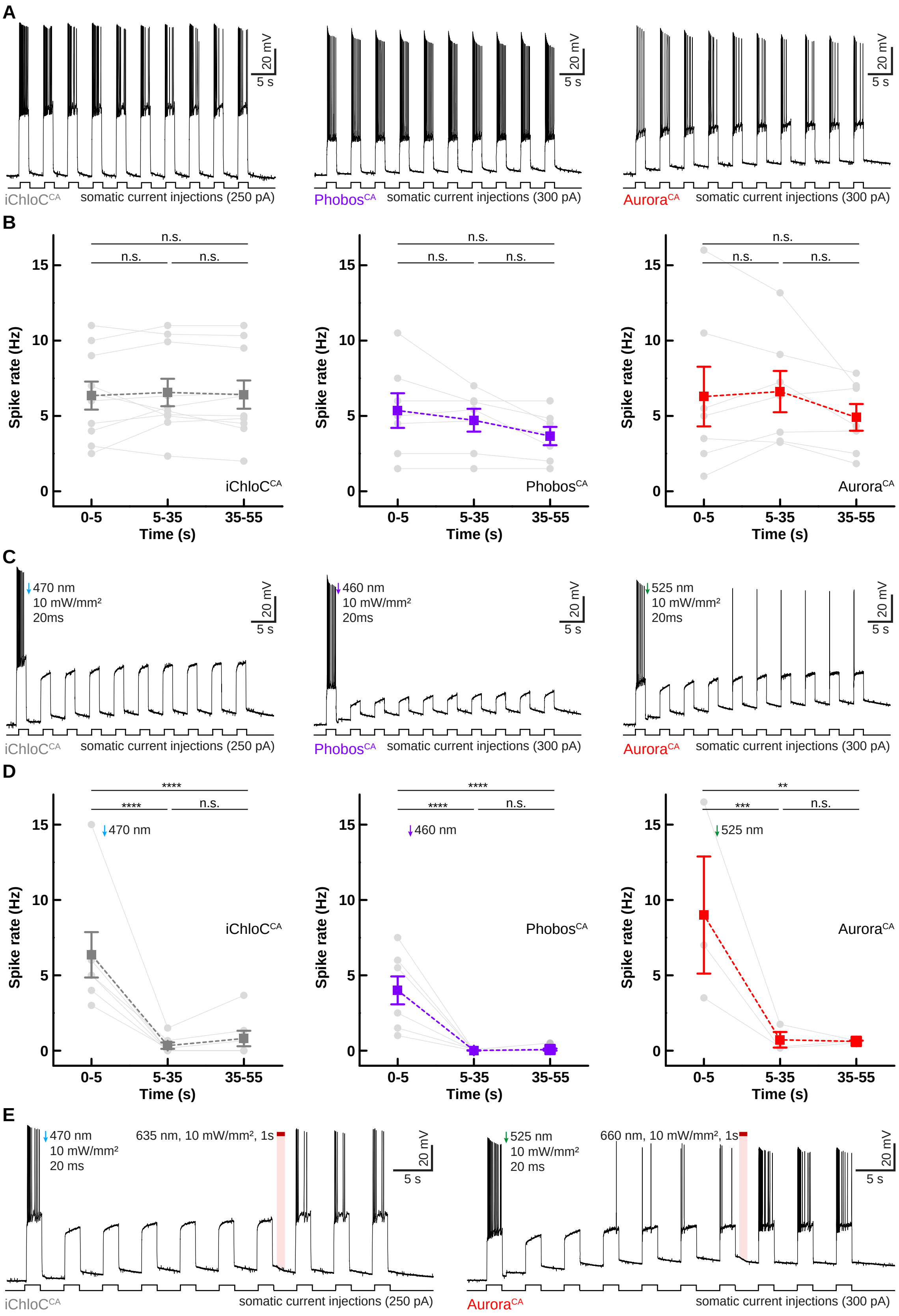

Figure S7: Performance of SFO-eACRs in CA1 pyramidal neurons. (**A**) Membrane voltage traces show reliable depolarization-induced action potential firing of Phobos^{CA}-Citrine, iChloC^{CA}-Citrine and Aurora^{CA}-Citrine expressing CA1 pyramidal neurons in the absence of light. (**B**) Quantification of the spike rate during current injection at indicated time intervals (n= 10, 7, 5 for iChloC^{CA}, Phobos^{CA} and Aurora^{CA}, respectively, repeated measures one-way ANOVA followed by Tukey's multiple comparisons test). (**C**) Membrane voltage traces show inhibition of depolarization-induced action potential firing of Phobos^{CA}-Citrine, iChloC^{CA}-Citrine and Aurora^{CA}-Citrine expressing CA1 pyramidal neurons in response to a 20 ms light pulse. (**D**) Quantification of the spike rate during current injection at indicated time intervals before (0-5 s) and after the light pulse (5-35 and 35-55 s). N= 7, 7, 3 for iChloC^{CA}, Phobos^{CA} and Aurora^{CA}, respectively, **: $p < 0.01$, ***: $p < 0.001$, ****: $p < 0.0001$, repeated measures one-way ANOVA followed by Tukey's multiple comparisons test. Light gray symbols indicate individual experiments. Averages are shown as rectangular symbols ± SEM. (**E**) Membrane voltage traces showing reversible suppression of depolarization-induced spiking by photoswitching iChloC^{CA} (left) and Aurora^{CA} (right) between open and closed state.

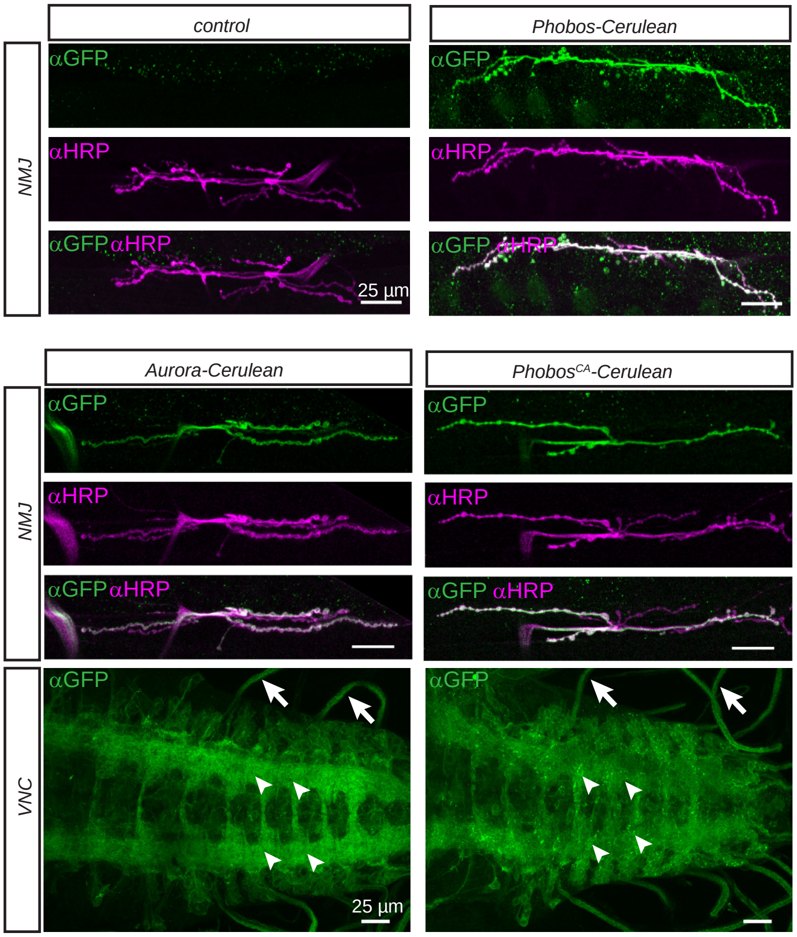

Figure S8: Expression and localization of Aurora, Phobos and Phobos^{CA}. Representative images of neuromuscular junctions (NMJs) at muscle 6/7 for control, Aurora-Cerulean, Phobos-Cerulean and Phobos^{CA}-Cerulean expressing motor neurons (*ok371-Gal4*). eACR expression and localization was visualized by anti-GFP immunohistochemistry together with a neuronal surface marker anti-HRP (horseradish peroxidase). eACRs did not obviously affect NMJ morphology and showed extensive colocalization with αHRP at the neuronal cell surface. Larval brain preparations show dendritic (arrowheads) and axonal (arrows) localization of Aurora-Cerulean and Phobos^{CA}-Cerulean in motor neurons of the ventral nerve chord (VNC). Phobos^{CA} expression resulted in some aggregation in soma and dendritic regions.

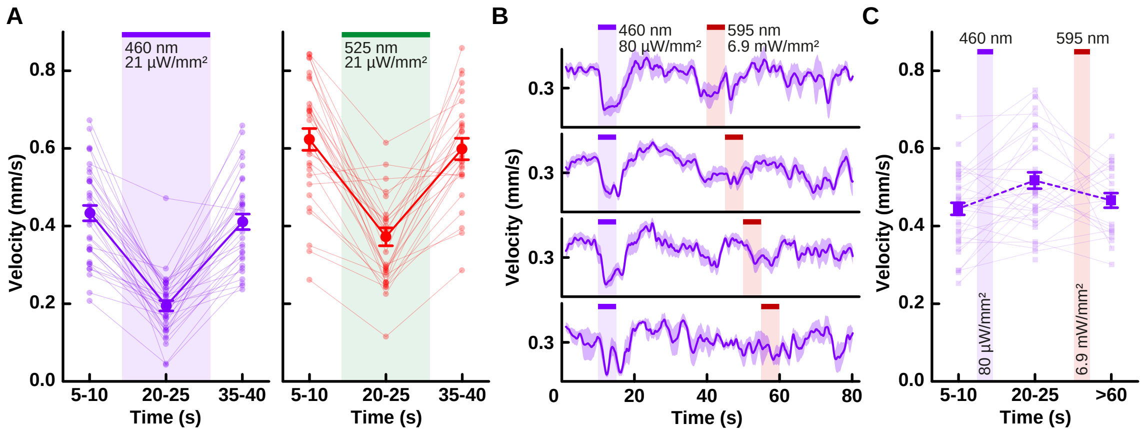

Figure S9: Innate responses to light during Drosophila locomotion behavior. (**A**) Averaged velocity of control animals (*w*⁻), with 15 s of light stimulation with 460 nm (left) or 525 nm (right). Data are shown as 5 s time bins before (5-10 s), during (20-25 s) and after (35-40 s) exposure to the indicated light intensity and wavelength (n= 37 for 460 nm and n= 34 for 525 nm, mean ± SEM). (**B**) Velocity of control animals (*w*⁻) over time exposed to 5 s of 460 nm light and 5 s of 595 nm light (same conditions as Phobos^{CA} animals in main figure 7C, n= 38, mean ± SEM). (**C**) Averaged velocity from experiments in (B) analyzed in 5 s time bins before (5-10 s), during (20-25 s) and after (>60 s) exposure to the indicated light intensity and wavelength (n= 37, mean ± SEM).

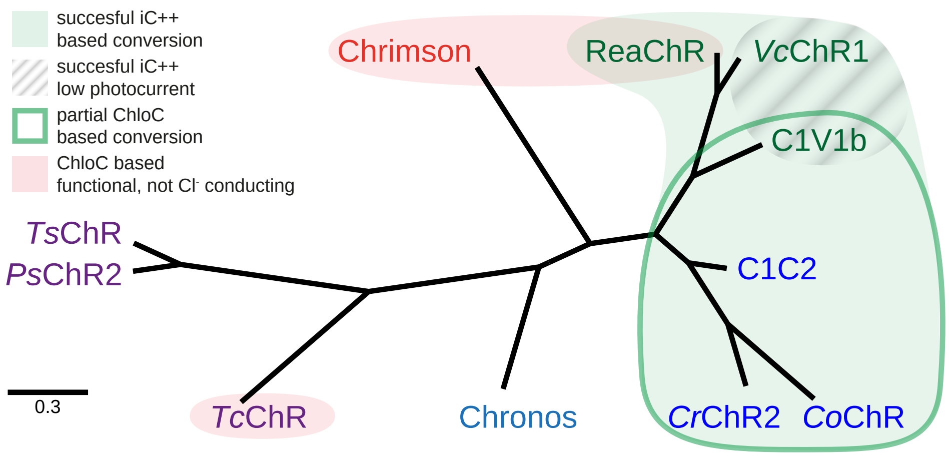

Figure S10: Phylogenetic tree of converted CCRs. A phylogenetic tree was generated for all CCRs listed in Figure S1 using phylogeny.fr[6]. N-terminal amino acids were excluded up to the position where the N-termini were replaced for the chimeric C1C2 N-terminus of iC++. Successfully converted CCRs cluster in the two right clades. CCR names are color coded according to their spectral absorption.

Table S1. Neuronal membrane parameters in the dark.

	wt (n = 10)	SD	Aurora (n = 9)	SD	Phobos (n = 7)	SD	Aurora^{CA} (n = 11)	SD	Phobos^{CA} (n = 8)	SD	iChloC^{CA} (n = 9)	SD	ANOVA	Stat. power
AP threshold (mV)	-50.70	2.47	-46.45	4.17	-44.99	3.73	-42.79	8.06	-47.48	4.29	-46.15	4.89	**	0.95
AP peak (mV)	35.82	2.94	32.31	2.47	34.49	3.08	35.82	4.15	32.71	2.23	35.98	4.68	n.s.	0.83
AP amplitude (mV)	114.05	5.37	107.57	3.88	108.31	4.71	107.02	7.59	112.14	4.35	115.32	3.65	*	0.99
n AP's	13.70	2.93	17.00	2.26	11.29	2.19	8.45	5.25	13.50	3.81	15.33	3.77	*	1.00
E_{Rest} (mV)	-78.23	4.61	-75.26	2.86	-73.82	3.35	-72.95	6.22	-79.43	3.74	-79.34	3.97	*	0.99
R_m (Mohm)	152.17	67.28	143.40	66.03	147.76	66.62	71.93	18.24	124.25	20.64	172.09	42.10	*	1.00

Electrical parameters of untransfected (wt) pyramidal CA1 neurons and neurons expressing the indicated aACR-Citrine fusion construct together with mCerulean. Action potentials (APs) were evoked by a square current pulse (500 ms, 500 pA) in current clamp mode. Threshold, peak voltage and amplitude were calculated for the first AP. Membrane resistance (R_m) was measured in voltage clamp mode in response to a square voltage pulse (-5 mV, 100 ms). E_{rest} = resting membrane potential, S.D. = standard deviation. Right columns indicate p-values from one-way ANOVA and power of statistical test for each parameter. n.s. no significant differences, * $p < 0.05$. Gray field marks value significantly different to non-transfected (wt) neurons. All measurements were liquid junction potential corrected.

References

1 McWilliam, H. *et al.* Analysis Tool Web Services from the EMBL-EBI. *Nucleic Acids Res* **41**, W597-600, doi:10.1093/nar/gkt376 (2013).

2 Li, W. *et al.* The EMBL-EBI bioinformatics web and programmatic tools framework. *Nucleic Acids Res* **43**, W580-584, doi:10.1093/nar/gkv279 (2015).

3 Sievers, F. *et al.* Fast, scalable generation of high-quality protein multiple sequence alignments using Clustal Omega. *Mol Syst Biol* **7**, 539, doi:10.1038/msb.2011.75 (2011).

4 Rajasethupathy, P. *et al.* Projections from neocortex mediate top-down control of memory retrieval. *Nature* **526**, 653-659, doi:10.1038/nature15389 (2015).

5 Berndt, A. *et al.* Structural foundations of optogenetics: Determinants of channelrhodopsin ion selectivity. *Proc Natl Acad Sci U S A* **113**, 822-829, doi:10.1073/pnas.1523341113 (2016).

6 Dereeper, A. *et al.* Phylogeny.fr: robust phylogenetic analysis for the non-specialist. *Nucleic Acids Res* **36**, W465-469, doi:10.1093/nar/gkn180 (2008).
